# A severe case of refractory esophageal stenosis induced by nivolumab and responding to tocilizumab therapy

**DOI:** 10.1186/s40425-018-0481-0

**Published:** 2018-12-27

**Authors:** Alice Horisberger, Stefano La Rosa, Jean-Philippe Zurcher, Stefan Zimmermann, Francois Spertini, George Coukos, Michel Obeid

**Affiliations:** 1 Department of Medicine, Division of Immunology and Allergy, Lausanne University Hospital CHUV, rue du Bugnon 46, CH-1011 Lausanne, Switzerland; 2 Service of Clinical Pathology, Institute of Pathology, Lausanne University Hospital CHUV, rue du Bugnon 25, CH-1011 Lausanne, Switzerland; 3 Department of Oncology, Lausanne University Hospital CHUV, rue du Bugnon 46, CH-1011 Lausanne, Switzerland; 4 Ludwig Institute for Cancer Research, University of Lausanne, chemin des Boveresses 155, CH-1066 Epalinges, Switzerland; 5 Vaccination and Immunotherapy Center, Lausanne University Hospital CHUV, rue du Bugnon 17, CH-1011 Lausanne, Switzerland; 6 Medical school Pitié-Salpêtrière, Sorbonne University, 91 Boulevard de l’Hôpital, F-75013 Paris, France

**Keywords:** Checkpoint inhibitors, Immune-related adverse events, Nivolumab, PD-1, Esophageal stenosis

## Abstract

**Background:**

The prevalence of esophageal stenosis caused by immune checkpoint inhibitors in the context of induced immune mucositis and esophagitis is extremely rare.

**Case presentation:**

We report the case of a patient with stage IV pulmonary adenocarcinoma treated for 6 months with nivolumab who developed bilateral sterile conjunctivitis followed by oropharyngeal mucositis and esophagitis complicated by a severe esophageal stenosis. The laryngeal margin and hypopharyngeal mucosa appeared highly inflammatory with fibrinous deposits. Esophagogastroduodenoscopy revealed mucositis with a scar-like structure immediately below the upper esophageal sphincter with nonulcerative mucosa and an inflammatory aspect of the entire esophagus. No involvement of the stomach was observed. Oropharynx biopsies displayed marked lymphocytic T cell-infiltration with several foci of monocellular necrosis in the squamous epithelium. No morphologic evidence of adenocarcinoma and no signs of mycotic, bacterial or viral infection were noted. A blood sample revealed a discrete increase in the erythrocyte sedimentation rate (ESR) with no eosinophilia or leukocytosis. Liver and kidney function panel tests were normal. A thoracoabdominal CT scan reported no evidence of disease recurrence. Despite multiple boluses of methylprednisolone and high doses of prednisone continued for several months, the patient experienced very rapid symptomatological reappearance during three steroid tapering attempts and aggravation of his esophageal stenosis to an aphagic stage, requiring a nasogastric tube. This long course of high-dose corticosteroid treatment was complicated with osteoporosis-induced fractures with several spontaneous compressions of thoracolumbar vertebrae requiring an enlarged T10 to L5 cementoplasty. Anti-IL-6 blockade therapy with tocilizumab resulted in excellent clinical response, allowing the total resolution of the immune-related adverse events (irAEs) and leading to successful steroid tapering.

**Conclusions:**

Herein, we describe the first case of a patient who developed autoimmune mucositis and esophagitis complicated by a severe refractory esophageal stenosis induced during treatment by nivolumab, which completely resolved after personalized treatment with tocilizumab, suggesting a role of IL-6 blockade in the management of severe steroid refractory esophageal stenosis and more broadly in refractory immune-related adverse events.

## Background

Immune checkpoint inhibitors (CPIs) have brought oncology into a new era by improving the overall survival of several malignancies [1, 2]. Among them, advanced non-small cell lung cancer (NSCLC) has become a major indication for the use of inhibitors of programmed cell death 1 (PD-1) and its ligand (PD-L1). The PD-1/PD-L1 axis is a crucial mediator of immune homeostasis, preventing autoimmune processes in the physiological setting, but also used by cancer to escape cellular immunity [3]. By blocking this T-cell downregulator, the medical community has been facing an entirely new spectrum of drug-induced autoimmune diseases classically reported as immune-related adverse events (irAEs). Although some organ systems are predominantly involved depending on the CPIs used, any organ can be affected [4]. Lower gastrointestinal (GI) tract irAEs, such as diarrhea and colitis, are described in up to one-third of patients treated with inhibitors of cytotoxic lymphocyte associated protein 4 (CTLA-4), with almost 10% of events classified as grade ≥ 3 [5]; however, these irAEs are less frequent and severe with anti-PD1 therapies [6]. In contrast, upper GI tract involvement has been more frequently reported with PD-1 inhibitors, although it is far less common and still poorly characterized. Recently, three cases of severe upper GI tract irAEs have been reported, displaying either gastroesophagitis or mucositis [7–9]. Here, we present the case of an immune mucositis and pharyngitis complicated by severe esophageal stenosis developed during nivolumab treatment and refractory to multiple corticosteroid lines but treated successfully with personalized anti-IL-6 blockade therapy (tocilizumab mAbs). To our knowledge, this is the first case of nivolumab-induced esophageal stenosis subject to personalized tocilizumab treatment reported to date.

## Case report

A 67-year-old male patient diagnosed with stage IV pulmonary adenocarcinoma was first treated with 6 cycles of carboplatin and pemetrexed followed by a maintenance regimen. Progression occurred within fourteen month after the start of the initial treatment. Consequently, second-line nivolumab was initiated at a dose of 3 mg/kg every two weeks.

**Fig. 1:**
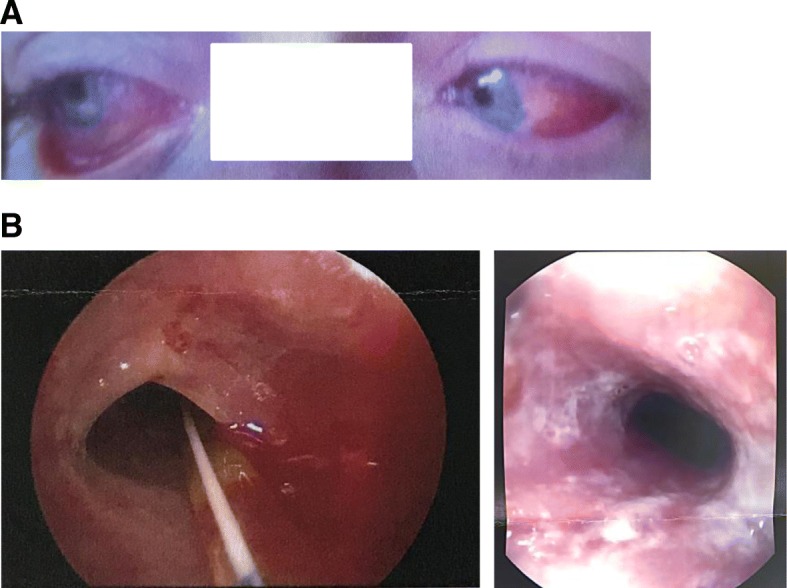
Patient bilateral conjunctivitis (**a**) and the esophageal stenosis, 1.5 cm immediately below the upper esophageal sphincter as observed by esophagogastroduodenoscopy (OGD) (**b**)

After thirteen doses of nivolumab, the patient complained of irritated red eyes without visual impairment. He did not exhibit skin involvement, arthralgia or urinary tract or digestive symptoms. The conjunctiva swab test was negative, and no improvement was observed with antibiotic ocular drops. The ophthalmologist’s examination revealed bilateral sterile conjunctivitis with no signs of uveitis or retinal lesions (Fig. 1a). The patient was treated with topical steroids with partial improvement.

A few days later, he developed fatigue and progressive dysphagia which became severe after two months followed by rapid 10-kg weight loss without symptoms of associated colitis or gastritis. At that point, the main differential diagnosis was esophageal infection, tumor progression with gastrointestinal (GI) upper tract involvement, paraneoplastic syndrome [10] or an atypical checkpoint inhibitor-related adverse event. Of note, the patient had no history of personal or familial autoimmune disease, conjunctivitis or upper digestive tract abnormality prior to nivolumab treatment. The oral examination initially revealed evidence for oral candidiasis, but treatment with a 7-day course of fluconazole did not improve dysphagia despite the resolution of the stomatitis. Bacterial culture of the oropharyngeal swab was negative, and PCR results for herpes simplex 1 and 2 infection were also negative. A blood sample revealed a discrete increase in the erythrocyte sedimentation rate (ESR) with no eosinophilia or leukocytosis. Liver and kidney function panel tests were normal. Thoracoabdominal CT scan reported no evidence of disease recurrence. Due to patient fatigue, a therapeutic break was implemented for one month with the introduction of prednisone at 30 mg per day with rapid tapering over 1 month. Although the patient initially experienced a partial resolution of dysphagia, a quick recurrence of symptoms was noticed as the prednisone dose was tapered. One month later, nivolumab was restarted, and the patient noticed a rapid deterioration of his dysphagia and mucositis. The clinical situation deteriorated despite the introduction of nystatin and steroid mouthwash, and the patient continued to lose weight. An esophagogastroduodenoscopy was done, which revealed diffuse mucositis with a scar-like stenosis immediately below the upper esophageal sphincter (Fig. 1b). Unlike the upper esophagus, the lower part and the stomach were of normal appearance.

**Fig. 2:**
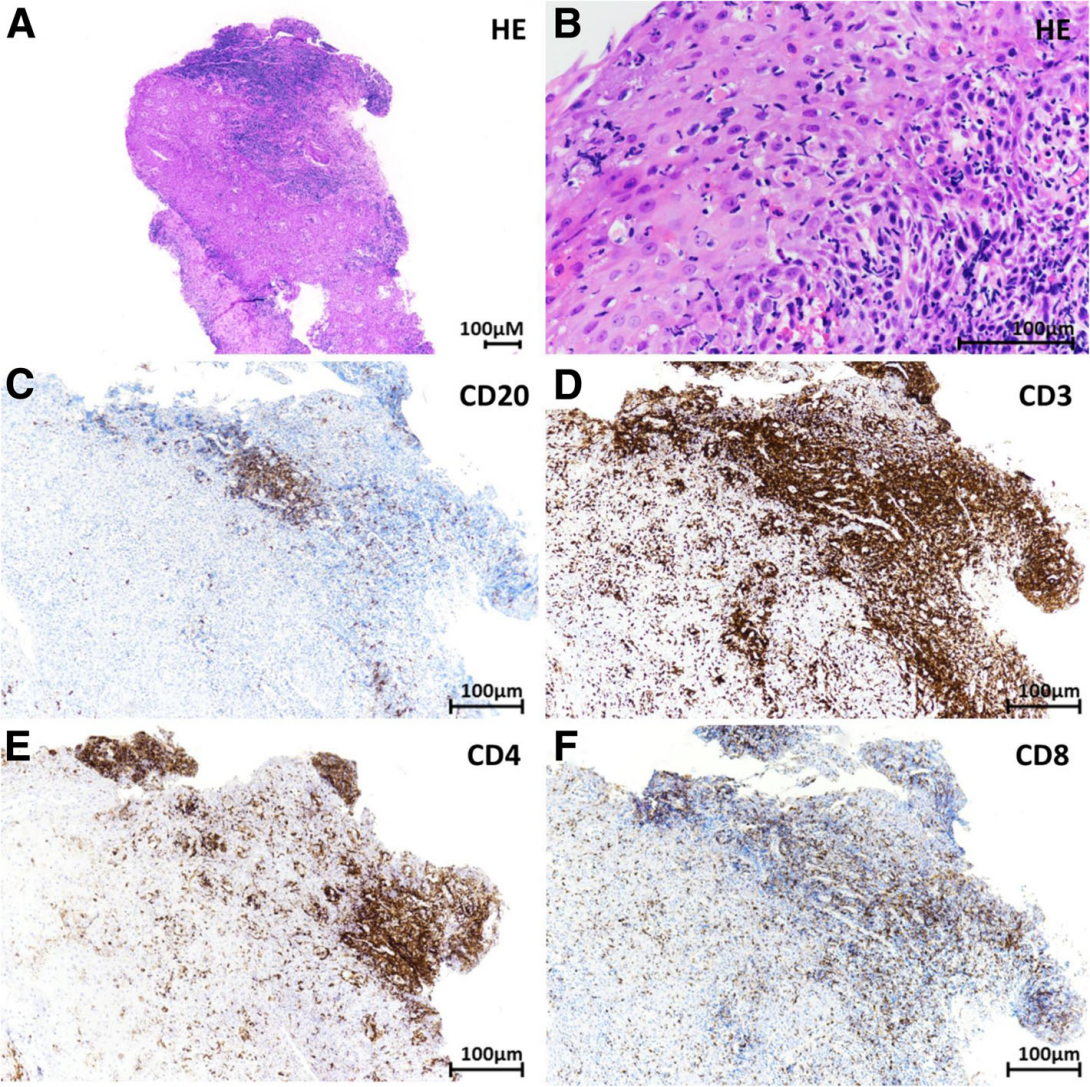
Morphological examination of oropharynx biopsies showed marked lymphocytic inflammation of the submucosa (**a**, original magnification × 40) with infiltration of the epithelial layer, where scattered apoptotic cells were observed (**b**, original magnification × 200). Immunohistochemical staining revealed that a minor population of lymphocytes (about 10%) was CD20 positive (**c**, original magnification × 100), while the majority of lymphocytic infiltration (about 90%) was represented by CD3-positive T-cells (**d**, original magnification × 100). Among T-lymphocytes, about 80% were CD4 positive (**e**, original magnification × 100) and about 20% were CD8 positive (**f**, original magnification × 100)

Biopsies of the oropharynx were performed, but esophageal dilatation was not performed due to the highly inflammatory mucosal status and patient anticoagulation. Faced with this significant loss of weight and the impossibility of an oral diet, a nasogastric tube was placed. Biopsies of the oropharynx displayed marked lymphocytic inflammation and several foci of monocellular necrosis in the squamous epithelium. Morphologic evidence of adenocarcinoma and signs of mycotic, bacterial or viral infection were not observed. Immunohistochemical stainings were performed in an automated stainer (Benchmark XT; Ventana Medical Systems, Tucson, AZ) using 3 μm-thick sections and the following antibodies: CD45 (monoclonal, clone 2B11 + PD7/26, Dako, Glostrup, Denmark), CD19 (monoclonal, clone BT51E, Novocastra, New Castle, UK), CD20 (monoclonal, clone L26, Novocastra), CD3 (monoclonal, 2GV6, Ventana), CD4 (monoclonal, SP35, Ventana), CD8 (monoclonal, C8/144B, Dako), CD68 (monoclonal, clone KP1, Dako), and PD-1 (polyclonal, R&D System, Inc., Minneapolis, MN, USA). Immunohistochemistry revealed a florid immune infiltrate, predominantly with T cells (90% CD45^+^CD19^−^CD20^−^CD3^+^), with only 10% of B cells (CD45^+^CD19^+^CD20^+^CD3^−^). Among T cells, the majority (80%) were CD4^+^. Few T cells expressed PD-1^+^. Rare macrophages were also observed (Fig. 2).

Based on these results, esophageal stenosis was considered a severe irAE secondary to an important mucosal inflammatory infiltrate. This severe dysphagia required enteral feeding, and nivolumab was permanently discontinued after seventeen doses. The patient was treated with 125 mg methylprednisolone followed by 1 mg/kg oral prednisone (total dose: 80 mg). After 3 days of treatment, the patient reported significant improvement, enabling him to eat solid food. Prednisone was tapered by 20 mg every two weeks until reaching a daily dose of 40 mg after 45 days of prednisone tapering, when he again presented increasing symptoms of severe dysphagia.

The patient was treated a second time with 125 mg methylprednisolone for 3 days followed by 1 mg/kg oral prednisone (total dose: 80 mg) with amelioration of dysphagia. Three weeks later, after the reduction of prednisone to 60 mg/daily, the patient noticed a new severe dysphagia deterioration, remaining aphagic. In this context, quick endoscopic esophageal dilatation was attempted. The laryngeal margin and hypopharyngeal mucosa appeared highly inflamed with fibrinous deposits on the direct laryngoscopy with no sign of salivary stasis or tumor invasion. Rigid esophagoscopy revealed erythema of the oropharynx with friable nonulcerative mucosa and an inflammatory aspect of the entire esophagus. Concomitant high-dose steroids at a dose of 125 mg of methylprednisolone for 3 days followed by 1 mg/kg prednisone again allowed transient symptomatic improvement.

One month later, recurrent dysphagia did not permit steroid tapering under 50 mg/d. Furthermore, the long course of high-dose corticosteroid treatment was complicated with severe osteoporosis and several spontaneous compression fractures of thoracolumbar vertebrae. Pathologic fracture due to metastases was ruled out by bone biopsy.An enlarged T10 to L5 vertebral cementoplasty was carried out. One month later due to a new episode of recurrent major dysphagia, the patient received a new bolus of 125 mg of methylprednisolone for 3 days followed by 1 mg/kg prednisone. The serum level of IL-6 was 3.10 pg/ml (normal range < 1.5 pg/ml), measured the same day before tocilizumab administration. Serum levels of IL-6 were assessed by electrochemiluminescence (ECL) Elecsys® IL-6 (Roche; Switzerland) according to the manufacturer instructions.

**Fig. 3:**
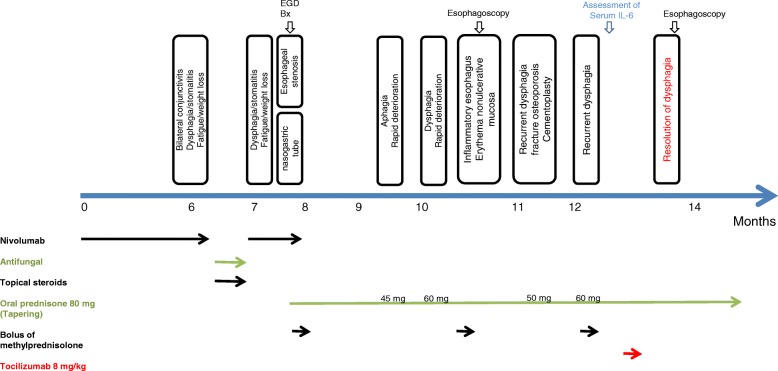
Patient timeline chart along with key dates for clinical manifestations, specific treatments and investigations. The dose of prednisone (PDN) labelled is the one at which the dysphagia relapse took place before the introduction of tocilizumab. PDN = prednisone, EGD = esophagogastroduodenoscopy, Bx = Biopsy of the oropharynx

At this point, a second line of immunosuppressive treatment was considered. Based on the oropharynx biopsy, histological analysis and the presence of a predominantly T-cell infiltrate, a single intravenous administration of the interleukin 6 receptor (IL-6R) neutralizing antibody tocilizumab at a dose of 8 mg/kg was given. This led to rapid amelioration of the symptomatology, with successful prednisone tapering without recurrent dysphagia. At the present time, 3 months after the administration of tocilizumab, the patient has experienced no relapse of dysphagia. A recent rigid endoscopy confirmed complete resolution of orolaryngopharyngeal and upper esophageal inflammation (Fig. 3).

During immunosuppressive therapy we observed no tumor progression. Eight months after discontinuing nivolumab treatment, the patient remains in complete remission with no radiographic evidence of tumor relapse.

## Discussion

Clinicians are progressively confronted with new types of irAEs with increasingly pleomorphic presentation [11]. Herein, we report the case of a patient displaying a severe and atypical upper GI tract irAE related to nivolumab immunotherapy. Oral mucositis and upper digestive tract irAEs are likely underestimated in clinical trials due to a lack of reporting given their primarily low-grade presentations. Mild stomatitis and mucositis have been reported in 5 to 9% of patients treated with nivolumab or pembrolizumab [6, 12] in prospective trials. A case of severe mucositis and esophagitis with histological documentation was reported in a patient receiving dose-escalated pembrolizumab (200 mg/arm every 3 weeks) in the Keynote 012 trial [8]. The endoscopic presentation matched ulcerative mucositis. Another patient treated with pembrolizumab for thymoma displayed severe cutaneo-mucositis, including esophagitis, mimicking Steven-Johnson syndrome [9]. The third case of severe upper GI tract irAE described in the literature involved a patient with Hodgkin’s lymphoma presenting diffuse esophagitis and gastritis secondary to nivolumab treatment [7]. All three patients presented heterogeneous clinical patterns, and no case involving esophageal stenosis has been described in the literature to date. Due to the close anatomical proximity of these mediastinal malignancies with the upper GI tract, contiguous collateral inflammation secondary to immune-checkpoint response is not excluded [13]. In contrast, our patient is the only reported patient with a tumor localized at distance from the involved upper GI tract.

The pathophysiology of irAEs is related to the loss of immune homeostasis, although the precise mechanism remains incompletely characterized. Interestingly, upper GI tract irAEs are mostly described with PD-1 inhibitors in contrast to lower GI tract irAEs, which are more prevalent with CTLA-4 inhibitors. These differences highlight the probability of distinct functions of CTLA-4 and PD-1 in gut immune-homeostasis [4]. Given the increased frequency and severity of ipilimumab-induced colitis, research efforts have been more extensively focusing on these agents. CTLA-4 plays a major role in microbiota immune-tolerance, and reciprocally its expression seems also to be influenced by the quality of the gut flora. In contrast, further research is needed to evaluate the role of PD-1 in the oropharynx and esophageal immune homeostasis. Recent reports suggest that the upper mucosal flora is less sensitive and thus more stable compared with its lower digestive counterpart [14]. The impact of chemotherapy and antibiotics on the lower GI tract microbiota is more pronounced given its increased bacterial load compared with the stomach and esophagus as well as the fact that the oral microbiota is less qualitatively sensitive to these agents [15]. These elements could explain the differences between the incidence of upper and lower GI tract irAEs. The occurrence of dysbiosis may stimulate the immune system, inducing a significant increase in immune activity in patients treated with CPIs. Interestingly, gut colonization with *Klebsiella pneumonia* isolated from the salivary microbiota in patients with Crohn’s disease induces Th1-driven inflammation in inoculated germ-free mice [16]. In our patient, we cannot exclude that the occurrence of concomitant oral candidiasis may have participated in triggering this irAE.

The clinical course of our patient was very challenging, with recurring severe symptomatic stenosis of the upper esophagus upon small steroid tapers, requiring each time boluses of methylprednisolone and increased steroid doses. Several agents have been proposed for the management of steroid-refractory or steroid-dependent irAEs, including antibodies blocking tumor necrosis factor alpha (TNFα) or mycophenolate mofetil, but the two molecules have not been approved by the patient’s insurance.

IL-6 is a principal acute inflammatory phase mediator that plays a major role in cytotoxic T-cell differentiation and activation and also exhibits protumor properties [17, 18]. Thus, the use of an IL-6 blockade strategy is particularly interesting given that it offers the advantage of a double effect without potentially compromising the efficacy of immunotherapy. Very interestingly, the combined blockade of IL-6 and PD-1/PD-L1 axe provides the synergistic effects not only on CD4+ Th1 response but also on the recruitment and function of CD8+ T cells in the tumor and its microenvironment [19, 20]. Moreover, the lack of interleukin-6 in the tumor microenvironment augments type-1 immunity and increases the efficacy of cancer immunotherapy [21].

After a single administration of tocilizumab, our patient showed an excellent response, allowing steroid tapering. Importantly, Stroud et al. proposed tocilizumab as a second-line therapy for irAEs [22]. Clinical improvement was observed in 79.4% of patients, with 52.9% of the patients requiring only a single dose for symptomatic response. Thereby, Stroud et al. proposed tocilizumab as a second-line therapy for steroid-refractory irAEs.

It is important to point out that pathogenic pro-inflammatory IL-17A-expressing CD4^+^ T cells subset (c-Kit^−^CD161^+^MDR1^+^ Th17 cells) have been reported to be key effectors of autoimmune inflammation refractory to glucocorticoids [23], which might suggest a role for this Th17 subset in steroid refractory irAEs. Importantly, IL-6 induces the development of Th-17 cells from naïve CD4+ T cells [24]. Thus, the IL-6 - Th-17 pathway could play a major role in the pathogenesis of irAEs, especially in steroid refractory cases.

This case report supports the use of anti-IL-6 therapy in complicated irAEs with unsatisfactory response to steroids as well as the rationale to use the predominant type of immune infiltrate on the biopsy (in this case T cells) as a biomarker to personalize treatment in steroid-refractory irAEs as we had just proposed in our recent therapeutic personalized algorithm based on selective inhibition of key inflammatory components involved in the pathophysiological processes of irAE without compromising cancer immunotherapy efficiency [25].

In our patient, the serum level of IL-6 was discreetly high, which is frequently observed in cancer patients [26]. Unfortunately, we do not have a kinetics of IL-6 serum concentrations to follow the temporal variation of IL-6 in our patient throughout this long period of irAEs and more particularly during corticosteroid therapy.

Between 10 and 20% of patients treated with PD-1 inhibitors develop unpredictable severe complications. At this time, no risk factor has been identified to predict severe irAEs, although some baseline aspects have been noted [27]. Autoimmunity risk is associated with personal or familial history of autoimmune disease; tumor location; and previous history of infections such as HIV, or concomitant medications. These factors have been proposed by Champiat et al. as predisposing factors for the development of irAEs [28]. Few baseline biomarkers have been identified in ipilimumab-treated melanoma patients, such as increased circulating eosinophil count, increased IL-17 blood levels and neutrophil infiltration of the colon lamina propria [29]. Recently, Gowen et al. observed a treatment-specific autoantibody signature using a proteomic microarray approach in the baseline serum from a subset of metastatic melanoma patients who developed severe irAEs [30]. These potential predictive biomarkers and their specificity for CTLA-4 and PD-1 blockade must be further examined in extended studies to confirm previous results and potentially guide immunotherapy management.

To the best of our knowledge, this is the first case report to detect intraoropharyngeal T cell infiltration followed by durable tumor response during PD-1 blockade therapy. Further studies may reveal whether tocilizumab could be considered also as secondary prevention, allowing to resume ICIs following irAEs.

## Abbreviations

CPIs: Checkpoint inhibitors
CTL4: Cytotoxic T-lymphocyte-associated protein 4
ESR: Erythrocyte sedimentation rate
GI: Gastrointestinal
irAEs: Immune-related adverse events
NSCLC: Non-small cell lung cancer
PD-1: Programmed cell death 1
PD-L1: Programmed cell death-ligand 1
